# Differentiating Well-Differentiated from Poorly-Differentiated HCC: The Potential and the Limitation of Gd-EOB-DTPA in the Presence of Liver Cirrhosis

**DOI:** 10.3390/diagnostics14151676

**Published:** 2024-08-02

**Authors:** Andrea Goetz, Niklas Verloh, Kirsten Utpatel, Claudia Fellner, Janine Rennert, Ingo Einspieler, Michael Doppler, Lukas Luerken, Leona S. Alizadeh, Wibke Uller, Christian Stroszczynski, Michael Haimerl

**Affiliations:** 1 Department of Radiology, University Hospital Regensburg, 93053 Regensburg, Germany; 2 Department of Diagnostic and Interventional Radiology, Medical Center University of Freiburg, Faculty of Medicine, University of Freiburg, 79085 Freiburg, Germany; 3 Department of Pathology, University Regensburg, 93053 Regensburg, Germany; 4 Department of Radiology, Klinikum Würzburg Mitte, 97074 Würzburg, Germany; 5 Department of Diagnostic and Interventional Radiology, University Hospital Frankfurt, 60596 Frankfurt am Main, Germany

**Keywords:** hepatocellular carcinoma (HCC), gadolinium ethoxybenzyl-diethylenetriaminepentaacetic acid (Gd-EOB-DTPA), magnetic resonance imaging (MRI), liver cirrhosis, tumor differentiation

## Abstract

This study uses magnetic resonance imaging (MRI) to investigate the potential of the hepatospecific contrast agent gadolinium ethoxybenzyl-diethylenetriaminepentaacetic acid (Gd-EOB-DTPA) in distinguishing G1- from G2/G3-differentiated hepatocellular carcinoma (HCC). Our approach involved analyzing the dynamic behavior of the contrast agent in different phases of imaging by signal intensity (SI) and lesion contrast (C), to surrounding liver parenchyma, and comparing it across distinct groups of patients differentiated based on the histopathological grading of their HCC lesions and the presence of liver cirrhosis. Our results highlighted a significant contrast between well- and poorly-differentiated lesions regarding the lesion contrast in the arterial and late arterial phases. Furthermore, the hepatobiliary phase showed limited diagnostic value in cirrhotic liver parenchyma due to altered pharmacokinetics. Ultimately, our findings underscore the potential of Gd-EOB-DTPA-enhanced MRI as a tool for improving preoperative diagnosis and treatment selection for HCC while emphasizing the need for continued research to overcome the diagnostic complexities posed by the disease.

## 1. Introduction

Hepatocellular carcinoma (HCC) presents a substantial global health challenge as the predominant form of primary liver cancer. Particularly prevalent in individuals with pre-existing cirrhosis, it ranks as the fifth most common malignancy in males and ninth in females with a rising incidence [1,2,3,4].

Given its asymptomatic nature in the early stages, HCC often eludes early detection, resulting in a diagnosis at advanced stages when curative therapy becomes infeasible, mainly as prognosis is largely dependent on the tumor stage and metastasis [5]. Therefore, developing robust and efficient strategies for early detection in at-risk populations is paramount in HCC management. HCC mostly occurs in patients with severe liver fibrosis or cirrhosis mainly due to infections with hepatitis B or hepatic C viruses as well as chronic alcohol abuse [6]. However, HCC also develops in patients with non-alcoholic fatty liver disease, hemochromatosis, or alpha-1 antitrypsin deficiency [4].

Current practices for early detection primarily involve semiannual ultrasound examinations for high-risk individuals. Suspicious findings from these examinations typically lead to further investigation using computed tomography (CT) or magnetic resonance imaging (MRI) [7,8]. While these methods have proven somewhat effective, there is ongoing debate regarding their relative efficiencies and potential for optimization.

Regarding the diagnostic pathway for uncertain liver lesions, lesions suspicious for malignancy and smaller than 1 cm should undergo sonographic follow-up in three months. For lesions exceeding 1 cm, further CT or MRI diagnostic imaging is recommended. Several studies have asserted the superiority of contrast-enhanced MRI over dynamic contrast-enhanced CT for detecting and differentiating liver lesions [9,10]. Upon arterial hypervascularization and portal venous washout observation, HCC diagnosis is considered validated [11]. In unclear cases, a biopsy is recommended [12].

Several studies have demonstrated that contrast-enhanced MRI is more sensitive in detecting HCC compared to dynamic contrast-enhanced CT. This increased sensitivity is likely due to the superior contrast delineation between the lesion and liver, as well as a more nuanced presentation of different tissue properties [9,10,13].

In clinical practice, liver MRI using the hepatocyte-specific contrast agent Gd-EOB-DTPA has become essential for the evaluation of malignant liver lesions.

By using this hepatocyte-specific contrast agent, diagnostic accuracy could be improved by performing an additional hepatobiliary late phase 20 min after contrast agent application [14].

The uptake of Gd-EOB-DTPA relies on functioning hepatocytes through organic anion transporters (OATP1B1/B3) and is excreted via the biliary tract through multidrug resistance-associated protein 2 (MRP2) [15,16].

A typical assessment of HCC involves the qualitative evaluation of signal intensity and the relative enhancement ratios, comparing the Gd-EOB-DTPA uptake in liver lesions to that in the surrounding liver parenchyma. However, the uptake of Gd-EOB-DTPA is also influenced by the presence and severity of cirrhosis [17], showing less Gd-EOB-DTPA uptake in cirrhotically remodeled liver parenchyma. Additionally, as the degree of differentiation of HCC increases, the number of hepatocytes expressing OATP1B1/B3 decreases and MRP2 increases, therefore leading to a reduced accumulation of Gd-EOB-DTPA compared to normal liver parenchyma [16,18]. As HCCs commonly occur in cirrhotic livers with impaired Gd-EOB-DTPA uptake, this assessment can be challenging.

Certain studies have also identified distinct enhancement patterns that could potentially correlate with different HCC stages [16,19,20,21,22]. However, there remains considerable ambiguity and controversy regarding whether these patterns reliably vary according to tumor grade.

The role of liver cirrhosis in influencing the diagnostic accuracy of these imaging techniques remains contentious and underexplored.

In this context, the primary objective of this study is to investigate whether specific enhancement patterns correspond to different grades of HCC. We also seek to examine the impact of liver cirrhosis on the diagnostic accuracy of these imaging strategies. Through this dual-pronged investigation, we hope to clarify some of the existing ambiguities in the field and contribute to the ongoing efforts to refine imaging strategies for early HCC detection.

## 2. Materials and Methods

### 2.1. Data Collection and Inclusion Criteria

This retrospective study was approved by the local institutional review board, ensuring that all the regulations and guidelines were followed. Our study incorporated patients who had undertaken Gd-EOB-DTPA-enhanced MRI of the liver before liver resection and had a histopathological confirmed, untreated, HCC within three months from the examination.

The initial data set comprised 59 resected HCC lesions derived from 52 patients. Exclusion criteria were respiratory artifacts (*n* = 4), incomplete detection of contrast medium phases (*n* = 3), infiltrative growth of the tumor (*n* = 4), or pre-treatment (*n* = 1). Thus, we inspected 47 lesions from 40 patients for their dynamic contrast behavior. Based on differentiation grade, 18 were classified as G1, 23 as G2, and 6 lesions as G3.

### 2.2. Imaging

Liver MRI scans were procured using a clinical whole-body 3T system (MAGNETOM Skyra, Siemens Healthcare, Erlangen, Germany), using a composite of body-spine array coil elements (comprising an 18-channel body matrix coil and a 32-channel spine matrix coil) for signal reception. The hepatospecific contrast agent was Gd-EOB-DTPA (Primovist^®^, Bayer Vital GmbH, Leverkusen, Germany). Each patient was administered a dose of Gd-EOB-DTPA, adapted according to their body weight (0.025 mmol/kg body weight). The administration was performed as a bolus injection at a flow rate of 1 mL/s, followed by a 20 mL NaCL flush. A T1-weighted volume interpolated breath-hold examination (VIBE) sequence with fat suppression was used for the signal intensity measurement. The specifications of this sequence are repetition time (TR) of 3.09 ms, echo time (TE) of 1.16 ms, a flip angle of 10°, parallel imaging factor of 2, 64 slices, a reconstructed voxel size of 1.3 × 1.3 × 3.0 mm, and a measured voxel size of 1.7 × 1.3 × 4.5 mm. This sequence spanned the entire liver and was captured in a single breath-hold before contrast injection (plain), during the arterial phase (AP) at 10 s, in the late arterial phase (LAP) at 40 s, in the portal venous phase (PVP) at 75 s, and in the hepatobiliary phase (HBP) at 20 min. The acquisition time for each VIBE sequence was 14 s.

### 2.3. Signal Intensity Measurement

Signal intensity (SI) was measured in liver lesions and adjacent liver tissue before and during various contrast medium phases, namely arterial, late arterial, portal venous, and hepatobiliary phase. One region of interest (ROI) was manually placed around the tumor boundary on the slice with the greatest tumor extension. This was done individually for each contrast medium phase.

For measuring the signal intensity of liver tissue, a second circular ROI was manually placed in the same slice in the liver parenchyma while avoiding any additional lesions or vascular structures.

The lesion contrast (C) was calculated for each liver lesion relative to the surrounding liver tissue using the formula:

C = (SI_Lesion_ − SI_Liver parenchyma_)/SI_Liver parenchyma_

where during the respective contrast phases, this measure demonstrated the contrast agent uptake ratio of the liver lesions to the surrounding liver tissue. It was correlated with the respective histopathological differentiation grade relative to each contrast medium phase.

### 2.4. Histopathological Analysis

For diagnostic purposes, tissue specimens were gathered from standard therapeutic surgeries conducted in a five-year period. The samples were preserved in neutral buffered formalin, followed by paraffin embedding. Tissue sections, 4 μm thick, were prepared following a conventional protocol and stained using hematoxylin and eosin (HE). A senior pathologist carried out histological evaluations. The grading of HCC was ascertained following the criteria laid down by the World Health Organization (WHO 5th edition) [23]. Based on the severity of malignancy, the HCCs were classified into three groups: G1, signifying well differentiated; G2, moderately differentiated; and G3, poorly differentiated. The fibrosis grade was graded using the Ishak scoring system [24]. In total, 26 HCC lesions were associated with incomplete or complete cirrhotic liver remodeling (ISHAK scores 5 and 6), whereas 21 lesions were not underpinned by cirrhosis (ISHAK scores 1–4).

### 2.5. Statistical Analysis

Statistical analysis was conducted using IBM SPSS Statistics Version 26 (Chicago, IL, USA). The measured signal intensities’ mean value and corresponding standard deviations were presented. Liver lesions were compared in terms of their degree of differentiation concerning contrast uptake using the non-parametric Mann-Whitney U test for independent variables. Statistical significance was set at *p* values < 0.05.

## 3. Results

### 3.1. Patient Demographics and Lesion Classification

In this study, we analyzed 47 HCC lesions from 40 patients. Well-differentiated HCC, denoted as G1 (*n* = 18), and poorly-differentiated HCC, combining G2 (*n* = 23) and G3 (*n* = 6) grades, were considered for analysis. The average age of the patients at the time of the MRI examination was 68.2 years, ranging from 44 to 83 years. No significant differences in the patients’ ages, body weights, heights, or BMIs were identified between the patients with normal liver function and those with liver cirrhosis. The majority of the patients were males (*n* = 35, 87.5%), while females constituted a minor proportion (*n* = 5, 12.5%). 26 HCC lesions (22 patients) were associated with precirrhotic or cirrhotic liver remodeling (ISHAK scores 5 and 6), whereas 21 lesions (18 patients) were not underpinned by cirrhosis (ISHAK scores 1–4). Table 1 provides a summary of the patient characteristics.

### 3.2. Qualitative Analysis of Signal Intensity

The signal intensities and contrast of HCC lesions were systematically evaluated against the surrounding liver parenchyma across distinct contrast medium phases. Mean values of the signal intensities for individual contrast medium phases with the corresponding lesion contrast are demonstrated in Table 2 and Table 3.

In the plain phase, the lesions’ average signal intensity (SI) was 168.85 ± 52.29, with a corresponding C of −0.09. When analyzed by differentiation grade, the G1 lesions had an SI of 169.00 ± 54.18 and a C of −0.01, while the combined G2/G3 lesions recorded an SI of 168.76 ± 52.05 and a C of −0.14. In the arterial phase, an increase in SI was observed. The average SI for all lesions was 251.68 ± 73.86, with a C of 0.25. The well-differentiated G1 lesions showed an SI of 260.11 ± 57.50 and a C of 0.40, whereas the combined G2/G3 lesions presented an SI of 246.45 ± 82.95 and a C of 0.17. In the late arterial phase, the average SI for all lesions was 266.91 ± 68.06, with a C of −0.07. The G1 lesions showed an SI of 283.83 ± 63.13 and a C of 0.03, while the G2/G3 lesions showed an SI of 256.41 ± 69.94 and a C of −0.12. In the portal venous phase, the average SI for all lesions was 265.15 ± 67.63, with a C of −0.09. The G1 lesions had an SI of 267.78 ± 61.65 and a C of −0.04, while the G2/G3 lesions recorded an SI of 263.52 ± 72.10 and a C of −0.13. In the hepatobiliary phase, the average SI for all lesions dropped to 235.34 ± 70.95 with a C of −0.25. The G1 lesions showed an SI of 246.33 ± 80.21 and a C of −0.19, whereas the G2/G3 lesions exhibited an SI of 228.69 ± 65.12 and a C of −0.29.

**Figure 1:**
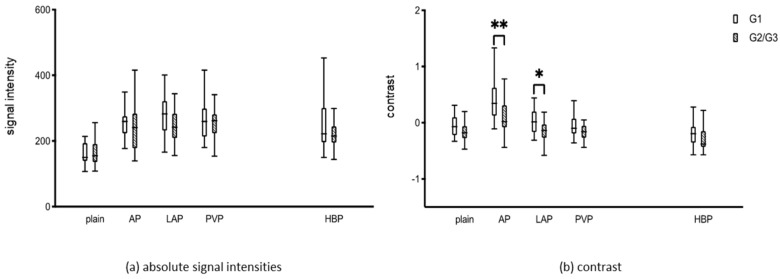
Contrast response of the liver lesions for the plain, arterial (AP), late atrial (LAP), portal venous (PVP), and hepatobiliary phase (HBP). (**a**) absolute signal intensities (**b**) contrast (C). *, *p* = 0.040; **, *p* = 0.010.

Figure 1a depicts the absolute signal intensities of liver lesions in each contrast medium phase and Figure 1b displays the C of HCC compared to adjacent liver parenchyma. A significant difference emerges between the well-differentiated and the G2/G3 lesions regarding the C in the arterial (*p* = 0.010) and late arterial phases (*p* = 0.040). However, no significant difference is noted in the subsequent phases. Both groups exhibit the typical HCC contrast behavior, characterized by arterial hypervascularization of the lesions and a washout of the contrast agent in the portal venous and hepatobiliary phases.

### 3.3. Influence of Liver Cirrhosis

The contrasting behavior was further investigated for the presence of liver cirrhosis. A total of 26 HCC lesions were associated with precirrhotic or cirrhotic liver remodeling (ISHAK scores 5 and 6), whereas 21 lesions were not underpinned by cirrhosis (ISHAK scores 1–4). For G1, the distribution was as follows: 8 lesions in patients without liver cirrhosis and 10 in patients with liver cirrhosis. For combined G2 and G3, the distribution was: 13 lesions in patients without liver cirrhosis and 16 in patients with liver cirrhosis.

**Figure 2:**
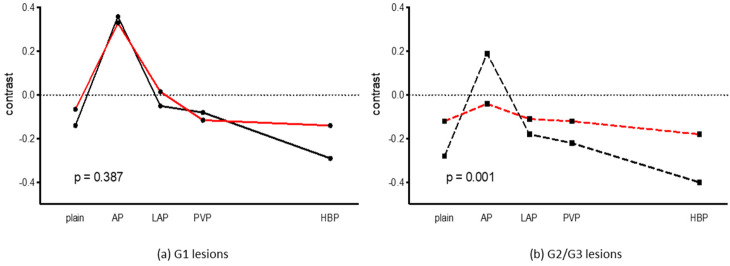
Contrast of HCC lesions in relation to cirrhotic (red) and non-cirrhotic (black) liver parenchyma for the plain, arterial (AP), late atrial (LAP), portal venous (PVP), and hepatobiliary phase (HBP). (**a**) G1. (**b**) G2/G3.

Liver cirrhosis had distinct implications for Gd-EOB-DTPA uptake into the surrounding liver parenchyma and the respective C of the HCC lesions. Figure 2 illustrates these impacts, showing that in a cirrhotically altered liver, there is a subtle change in C between the portal venous and hepatobiliary phase. In contrast, a more noticeable reduction in C is observed in healthy liver tissue. A significant difference was observed for G2/G3 tumors between non- cirrhotic and cirrhotic liver parenchyma (*p* = 0.001), whereas no significant difference was observed for G1 tumors.

### 3.4. Image Examples

**Figure 3:**
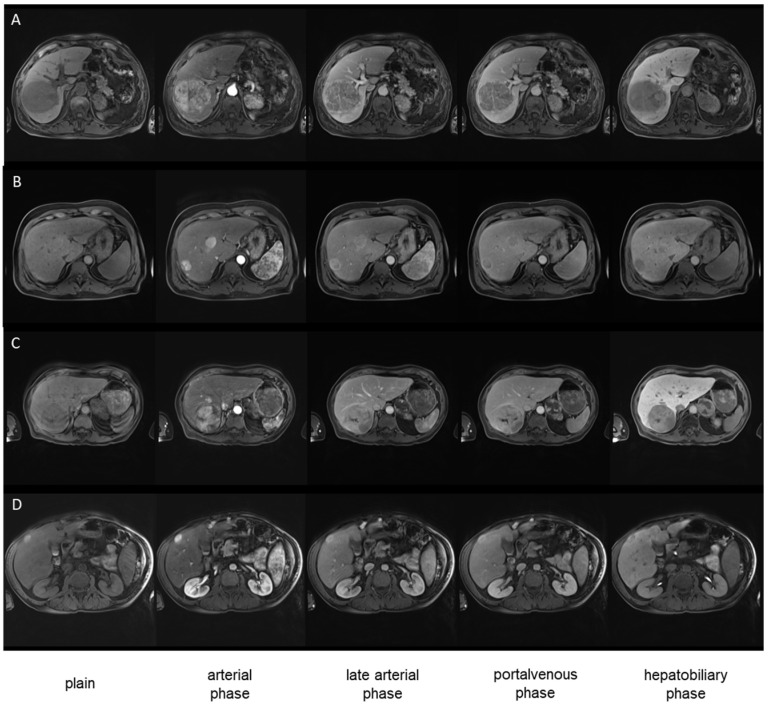
Comparison of well-differentiated and poorly-differentiated HCCs in the presence of liver cirrhosis (ISHAK Score 6) in T1 weighted VIBE sequences (phases as indicated): (**A**) well-differentiated HCC (G1) in liver fibrosis (ISHAK 2), (**B**) well-differentiated HCC (G1) in liver cirrhosis (ISHAK 6), (**C**) poorly-differentiated HCC (G2) in normal liver parenchyma (ISHAK 0), (**D**) poorly-differentiated HCC (G2) in in liver cirrhosis (ISHAK 6).

Examples of the dynamic contrast behavior of HCC lesions in non-cirrhotic and cirrhotic liver parenchyma are shown in Figure 3. All HCC lesions show the typical arterial hypervascularization with washout in the portal venous phase and hypointensity in the hepatobiliary phase. The uptake of Gd-EOB-DTPA in the surrounding liver parenchyma differs between non-cirrhotic and cirrhotic liver parenchyma, thus affecting the contrast of HCC lesions.

## 4. Discussion

To the best of our knowledge, this is the first study to investigate the impact of liver function on Gd-EOB-DTPA for diagnosing HCC in relation to its grading.

Preoperative determination of the degree of differentiation of HCC is essential for choosing appropriate therapy [25,26,27,28]. Various agents like atorvastatin [29], cefazolin [30], and chemotherapeutic agents such as methotrexate [31], rifampicin [32], paclitaxel, and docetaxel [33] are also absorbed into cells via OATP 1B1 and 1B3. This suggests that tumor therapy effectiveness may depend on tumor differentiation degree as it correlates with these transporter proteins’ expression.

According to current German guidelines, the treatment of HCC in a cirrhotic liver is liver transplantation, which also treats the underlying cirrhosis. However, due to organ shortage, there are strict regulations about the eligibility of liver transplantation such as the Milan criteria, in which patients are considered eligible for liver transplantation with one HCC lesion up to 5 cm or up to 3 HCC lesions between 1–3 cm (Mazzaferro 1996). Because these strict criteria may exclude potential patients who would benefit from liver transplantation, it has been suggested that the degree of differentiation should also play a role in the selection criteria for liver transplantation [25,26], as it has been shown that the histopathological degree of differentiation is a prognostic factor for the survival rate [27,28].

Due to this prognostic influence, patients with poorly-differentiated HCC are excluded from liver transplantation and treated palliatively in some centers [34,35]. Therefore, determining the correct degree of differentiation of the presenting HCC plays a significant role.

The histopathological degree of differentiation is determined by biopsy, usually as part of the primary diagnosis, with the most common procedure being punch biopsy, which can be associated with complications such as bleeding, infection, or carryover of tumor tissue [36]. Pawlik et al. [37] show a specificity of 92.5% and a sensitivity of only 34.6% concerning the determination of a low degree of differentiation (G3) so that a false-negative classification can frequently occur here.

Several studies have reported that HCCs’ appearance on Gd-EOB-DTPA-enhanced MRI examinations vary based on their grading [38,39,40,41,42,43]. However, several other studies contradict these results, asserting that Gd-EOB-DTPA uptake in HCC lesions does not correlate with differentiation degree [44,45,46,47]. The regulation of Gd-EOB-DTPA’s uptake and excretion is controlled by OATP 1B1/1B3 and MRP-2 [15]. OATPs are multispecific transporter proteins, with OATP 1B1 and 1B3 subtypes expressed explicitly in the liver [48]. Studies have demonstrated that advanced HCC lesions often exhibit reduced OATP 1B1 or 1B3 expression, while MRP2 expression remains stable or increases [16,18]. These findings corroborate our results, which suggest decreased Gd-EOB-DTPA accumulation in poorly-differentiated HCC lesions, potentially due to reduced Gd-EOB-DTPA uptake or increased biliary excretion rates. Frericks et al. [44] and Schelhorn et al. [39] showed no correlation between the difference in grade and the signaling of liver lesions. Still, histopathological grading was performed only on biopsy specimens and not completely resected liver tissue. In addition, the evaluation was based on signaling changes of the lesions compared with the surrounding liver parenchyma, without considering whether there was underlying cirrhosis and thus possibly impaired Gd-EOB- DTPA uptake. Tsuboyama et al. [46]. demonstrated overexpression of OATP—1B3 at all stages of differentiation, linking high Gd-EOB-DTPA accumulation of lesions to altered expression of MRP-2 and, thus, possibly, decreased excretion. They defined high Gd-EOB-DTPA enrichment as greater enrichment compared with surrounding liver parenchyma, independent of liver function. Since they listed only five lesions with high enhancement in their study, no conclusion could be drawn regarding the correlation between tumor grade and Gd-EOB-DTPA enhancement.

The presence of liver cirrhosis, one of the most critical risk factors for developing HCC, also influences Gd-EOB-DTPA uptake in the pre-damaged liver parenchyma [17]. Tamada et al. [49] showed that the accumulation of Gd-EOB-DTPA in the liver parenchyma is significantly reduced by liver cirrhosis, especially in the Child C stage, most likely due to a reduced number of healthy hepatocytes or impaired contrast agent uptake into liver cells. Therefore, marked liver cirrhosis shows a variable appearance of the liver in contrast-enhanced MRI examinations so that the hepatobiliary phase in liver cirrhosis can sometimes only be assessed to a limited extent because the surrounding liver parenchyma only accumulates suboptimally [50]. In MRI diagnostics, the signal behavior is generally considered qualitatively, and lesions are therefore assessed in hyper-, iso-, or hypointense, depending on the surrounding tissue. Similarly, the contrast of liver lesions used in this work is calculated using the signal intensity of the surrounding liver parenchyma. It is thus influenced by the condition of the surrounding liver parenchyma in case of liver fibrosis or cirrhosis.

In the case of impaired liver function, it is therefore not possible to clearly distinguish based on the lesion contrast whether it is due to reduced uptake of Gd-EOB-DTPA into the cirrhotically remodeled liver parenchyma or to washout of the liver lesions.

This study’s primary limitation is the small number of HCC lesions included, and the rarity of G3 lesions limited their inclusion to only six [51]. HCC’s high intratumoral heterogeneity also challenges pathologists and radiologists, as different degrees of differentiation may exist within one tumor [52].

## 5. Conclusions

This study emphasizes the potential of using the hepatospecific contrast agent Gd-EOB-DTPA in differentiating well-differentiated from poorly-differentiated HCC during MRI examinations. By analyzing the dynamic contrast agent behavior, we could distinguish between different stages of HCC differentiation, thereby contributing to the precision of preoperative diagnosis and selection of suitable therapeutic strategies. Further research is needed to confirm the utility of combining radiologic and histopathologic features in grading HCC, which could lead to more accurate therapeutic decisions.

However, our findings also highlight a potentially limited additional diagnostic value of the late hepatobiliary phase in cirrhotically remodeled liver parenchyma due to altered pharmacokinetics affecting the contrast agent behavior of HCC lesions. This illustrates the complexity of HCC diagnosis and underlines the importance of a comprehensive understanding of HCC and its behaviors in various physiological contexts.

## Figures and Tables

**Table 1:** Patient characteristics (NCL, non-cirrhotic liver parenchyma; LC, liver cirrhosis).

	All (*n* = 40)	NCL (*n* = 18)	LC (*n* = 22)
age (years)	68.2 (44–83)	67.3 (44–79)	68.8 (45–83)
gender			
- men, *n* (%)	35 (87.5%)	16 (88.9%)	19 (86.4%)
- women, *n* (%)	5 (12.5%)	2 (11.1%)	3 (13.6%)
height (m)	1.72 ± 0.09	1.72 ± 0.10	1.72 ± 0.09
weight (kg)	80.20 ± 16.63	78.59 ± 18.18	80.48 ± 16.37
BMI	27.13 ± 4.48	26.29 ± 4.99	27.18 ± 4.44

**Table 2:** Mean signal intensity (SI) by phase and differentiation grade.

	All (SI)	G1 (SI)	G2/G3 (SI)	*p*
plain	168.85 ± 52.29	169.00 ± 54.18	168.76 ± 52.05	0.95
arterial phase	251.68 ± 73.86	260.11 ± 57.50	246.45 ± 82.95	0.30
late arterial phase	266.91 ± 68.06	283.83 ± 63.13	256.41 ± 69.94	0.06
portal venous phase	265.15 ± 67.63	267.78 ± 61.65	263.52 ± 72.10	0.73
hepatobiliary phase	235.34 ± 70.95	246.33 ± 80.21	228.69 ± 65.12	0.39

**Table 3:** Lesion contrast (C) by phase and differentiation grade.

	All (C)	G1 (C)	G2/G3 (C)	*p*
plain	−0.09	−0.01	−0.14	0.08
arterial phase	0.25	0.40	0.17	0.01
late arterial phase	−0.07	0.03	−0.12	0.04
portal venous phase	−0.09	−0.04	−0.13	0.11
hepatobiliary phase	−0.25	−0.19	−0.29	0.14

## Data Availability

The data presented in this study are available on request from the corresponding author.
